# Supplementary material for: A transcriptional constraint mechanism limits the homeostatic response to activity deprivation in mammalian neocortex
Source: eLife. 2023 Feb 7;12:e74899. doi: 10.7554/eLife.74899 (PMC10010687; doi:10.7554/eLife.74899)
Supplement: Figure 7—source data 2. [file elife-74899-fig7-data2.pdf]

DKO_Survival

Isabel

2/24/2021

pdf_document: default html_document: default word_document: default —

Survival Curve:

```
library(tinytex)
library(tidyverse)
```

```
## -- Attaching packages --------------------------- tidyverse 1.3.0 --
```

```
## v ggplot2 3.3.2     v purrr   0.3.4
## v tibble  3.0.1     v dplyr   1.0.0
## v tidyr   1.1.0     v stringr 1.4.0
## v readr   1.3.1     v forcats 0.5.0
```

```
## -- Conflicts ------------------------------ tidyverse_conflicts() --
## x dplyr::filter() masks stats::filter()
## x dplyr::lag()    masks stats::lag()
```

```
library(survival)
colors1=c("red","blue","dark green","purple","black")
survival <- read.csv("C:/Users/isabe/OneDrive/Documents/Nelson Lab/survivalcurve3.csv")
print(survival)
```

```
##      ï.. time status   group
## 1      1   14      1 DBP het
## 2      2   25      1 DBP het
## 3      3   26      1 DBP het
## 4      4   37      1 DBP het
## 5      5   59      1 DBP het
## 6      6   60      1 DBP het
## 7      7   64      1 DBP het
## 8      8   72      1 DBP het
## 9      9   85      1 DBP het
## 10    10  120      1 DBP het
## 11    11  128      1 DBP het
## 12    12  151      1 DBP het
## 13    13  155      1 DBP het
## 14    14   51      0 DBP het
## 15    15   55      0 DBP het
## 16    16   60      0 DBP het
## 17    17   62      0 DBP het
## 18    18   68      0 DBP het
## 19    19   70      0 DBP het
## 20    20   71      0 DBP het
## 21    21   72      0 DBP het
## 22    22   84      0 DBP het
## 23    23   87      0 DBP het
## 24    24   91      0 DBP het
## 25    25   94      0 DBP het
## 26    26   97      0 DBP het
## 27    27   97      0 DBP het
## 28    28   98      0 DBP het
## 29    29  100      0 DBP het
## 30    30  106      0 DBP het
## 31    31  107      0 DBP het
## 32    32  107      0 DBP het
## 33    33  122      0 DBP het
## 34    34  124      0 DBP het
## 35    35  127      0 DBP het
## 36    36  127      0 DBP het
## 37    37  127      0 DBP het
## 38    38  131      0 DBP het
## 39    39  152      0 DBP het
## 40    40  176      0 DBP het
## 41    41   46      0 DBP het
## 42    42   45      0 DBP het
## 43    43   34      0 DBP het
## 44    44   34      0 DBP het
## 45    45   33      0 DBP het
## 46    46   33      0 DBP het
## 47    47   32      0 DBP het
## 48    48   30      0 DBP het
## 49    49   30      0 DBP het
## 50    50   29      0 DBP het
## 51    51   24      0 DBP het
## 52    52   23      0 DBP het
```

```
## 53    53   35      0 DBP het
## 54    54   33      0 DBP het
## 55    55   33      0 DBP het
## 56    56   29      0 DBP het
## 57    57   37      0 DBP het
## 58    58   37      0 DBP het
## 59    59   25      0 DBP het
## 60   196   21      1     TKO
## 61   197   24      1     TKO
## 62   198   69      1     TKO
## 63   199   32      1     TKO
## 64   200   20      1     TKO
## 65   201   87      1     TKO
## 66   202  101      1     TKO
## 67   203   20      1     TKO
## 68   204   35      1     TKO
## 69   205   74      1     TKO
## 70   206   28      1     TKO
## 71   207  123      1     TKO
## 72   208   43      1     TKO
## 73   209  134      1     TKO
## 74   210   20      1     TKO
## 75   211   45      1     TKO
## 76   212   23      1     TKO
## 77   213   93      1     TKO
## 78   214   20      1     TKO
## 79   215   20      1     TKO
## 80   216   20      1     TKO
## 81   217   20      1     TKO
## 82   218   98      1     TKO
## 83   219   17      1     TKO
## 84   220   17      1     TKO
## 85   221   23      1     TKO
## 86   222   18      1     TKO
## 87   223  148      0     TKO
## 88   224   70      0     TKO
## 89   225   59      0     TKO
## 90   226  150      0     TKO
## 91   227   93      0     TKO
## 92   228   54      0     TKO
## 93   229  104      1     TKO
## 94   230   82      1     TKO
## 95   231  127      1     TKO
## 96   232   26      1     TKO
## 97   233   26      1     TKO
## 98   234  155      0     TKO
## 99   235  148      0     TKO
## 100  236   43      0     TKO
## 101  237   34      0     TKO
## 102  238   28      0     TKO
## 103  239   18      0     TKO
## 104  240   46      0     TKO
## 105  241   42      0     TKO
## 106  242   40      0     TKO
```

```
## 107  243   38      0     TKO
## 108  244   28      0     TKO
## 109  245   27      0     TKO
## 110  246   27      0     TKO
## 111  247   24      0     TKO
## 112  248   24      0     TKO
## 113  249   24      0     TKO
## 114  250   24      0     TKO
## 115  251   22      0     TKO
## 116  252   22      0     TKO
## 117  253   19      0     TKO
## 118  254   19      0     TKO
## 119  255   16      0     TKO
## 120  256   14      0     TKO
## 121  257   10      0     TKO
## 122  258   28      0     TKO
## 123  259   27      0     TKO
## 124  260   27      0     TKO
## 125  261   27      0     TKO
## 126  262   25      0     TKO
## 127  263   22      0     TKO
## 128  264   21      0     TKO
## 129  265   29      0     TKO
## 130  266   23      1 HLF het
## 131  267   21      1 HLF het
## 132  268   92      1 HLF het
## 133  269  105      1 HLF het
## 134  270  204      0 HLF het
## 135  271  198      0 HLF het
## 136  272  170      0 HLF het
## 137  273  168      0 HLF het
## 138  274  154      0 HLF het
## 139  275  122      0 HLF het
## 140  276  120      0 HLF het
## 141  277   66      0 HLF het
## 142  278   59      0 HLF het
## 143  279   57      0 HLF het
## 144  280   55      0 HLF het
## 145  281   51      0 HLF het
## 146  282   73      0 HLF het
## 147  283  199      0 HLF het
## 148  284  174      0 HLF het
## 149  285  259      0 HLF het
## 150  286  194      0 HLF het
## 151  287  150      0 HLF het
## 152  288  140      0 HLF het
## 153  289  189      0 HLF het
## 154  290   33      0 HLF het
## 155  291   33      0 HLF het
## 156  292   26      0 HLF het
## 157  293   22      0 HLF het
## 158  294   21      0 HLF het
## 159  295   21      0 HLF het
## 160  296   21      0 HLF het
```

```
## 161  297   16      0 HLF het
## 162  298   15      0 HLF het
## 163  299   15      0 HLF het
## 164  300   28      0 HLF het
## 165  301   19      0 HLF het
## 166  302   86      0      WT
## 167  303   86      0      WT
## 168  304   80      0      WT
## 169  305   80      0      WT
## 170  306   80      0      WT
## 171  307   80      0      WT
## 172  308  102      0      WT
## 173  309  102      0      WT
## 174  310  200      0      WT
## 175  311   45      0      WT
## 176  312   45      0      WT
## 177  313   45      0      WT
## 178  314  136      0      WT
## 179  315  136      0      WT
## 180  316   23      0      WT
## 181  317   21      0      WT
## 182  318   21      0      WT
## 183  325  161      0     TKO
## 184  326  129      0     TKO
## 185  327   23      0     TKO
## 186  328   23      0     TKO
## 187  329   23      0     TKO
## 188  330   23      0     TKO
## 189  331   23      0     TKO
## 190  338   74      0     TKO
## 191  343  129      0     TKO
## 192  347   45      0      WT
## 193  348   45      0      WT
## 194  349   45      0      WT
## 195  350  136      0      WT
## 196  351  136      0      WT
## 197  352   27      1     TKO
## 198  353   27      1     TKO
## 199  354   19      1     TKO
## 200  355   20      1     TKO
## 201  356   21      1     TKO
## 202  357   25      1     TKO
## 203  358   25      1     TKO
## 204  359   17      1     TKO
## 205  360   18      1     TKO
## 206  362   22      1     TKO
## 207  363   27      1     TKO
## 208  365  158      0     TKO
## 209  366  190      0     TKO
## 210  374   40      0     TKO
## 211  383  154      0     TKO
## 212  388   63      1     TKO
## 213  390   94      1     TKO
## 214 2169  243      0 TEF het
```

```
## 215 2170  156      0 TEF het
## 216 2171  137      0 TEF het
## 217 2172  137      0 TEF het
## 218 2177  137      0 TEF het
## 219 2173   78      1 TEF het
## 220 1729  131      0 TEF het
## 221 1651  147      0 TEF het
## 222 1652  133      0 TEF het
## 223 2351  172      0 TEF het
## 224 2352  138      0 TEF het
## 225 2122  104      0 TEF het
## 226 2038  340      0 TEF het
## 227 2050  322      0 TEF het
## 228 2256  228      0 TEF het
## 229 2255  209      0 TEF het
## 230 2008  205      0 TEF het
## 231 2003  186      0 TEF het
## 232 2010  180      0 TEF het
## 233 2347  176      0 TEF het
## 234 2363  174      0 TEF het
## 235 2044  168      0 TEF het
## 236 2146  158      0 TEF het
## 237 2252  158      0 TEF het
## 238 2252  158      0 TEF het
## 239 2257  158      0 TEF het
## 240 2257  158      0 TEF het
## 241 2362  155      0 TEF het
## 242 1942  151      0 TEF het
## 243 1943  151      0 TEF het
## 244 2161  150      0 TEF het
## 245 2165  149      0 TEF het
## 246 2328  144      0 TEF het
## 247 2053  132      0 TEF het
## 248 1675  127      0 TEF het
## 249 2105  122      0 TEF het
## 250 2365  121      0 TEF het
## 251 2042  118      0 TEF het
## 252 2062  116      0 TEF het
## 253 2369  116      0 TEF het
## 254 2060  105      0 TEF het
## 255 2063  102      0 TEF het
## 256 2064  102      0 TEF het
## 257 2066  102      0 TEF het
## 258 2368  101      0 TEF het
## 259 2086   93      0 TEF het
## 260 2088   93      0 TEF het
## 261 2099   85      0 TEF het
## 262 2115   64      0 TEF het
## 263 2449   64      0 TEF het
## 264 2450   64      0 TEF het
## 265 2451   64      0 TEF het
## 266 2452   64      0 TEF het
## 267 2144   51      0 TEF het
## 268 2372   50      0 TEF het
```

```
## 269 1939  151      1 TEF het
## 270 2065   49      1 TEF het
## 271 2159   21      1 TEF het
## 272 2254  119      1 TEF het
## 273 2197  232      0 TEF het
## 274 2166  149      0 TEF het
## 275 2167  149      0 TEF het
## 276 2199  147      0 TEF het
## 277 1668  141      0 TEF het
## 278 2198  128      0 TEF het
## 279 2207  128      0 TEF het
## 280 1928   98      0 TEF het
## 281 1929   98      0 TEF het
## 282 2370   50      0 TEF het
## 283 2371   50      0 TEF het
## 284 2374   50      0 TEF het
## 285 2193   69      1 TEF het
## 286 1898  103      0 TEF het
## 287 1345  176      0 TEF het
## 288 2299  223      0 TEF het
## 289 2277  212      0 TEF het
## 290 2278  212      0 TEF het
## 291 2281  194      0 TEF het
## 292 2279  193      0 TEF het
## 293 2280  193      0 TEF het
## 294 2304  192      0 TEF het
## 295 2298  151      0 TEF het
## 296 2301  151      0 TEF het
## 297 2303  151      0 TEF het
## 298 2443  118      0 TEF het
## 299 2444  117      0 TEF het
## 300 2432  111      0 TEF het
## 301 2433  111      0 TEF het
## 302 2434  111      0 TEF het
## 303 2436   98      0 TEF het
## 304 2437   98      0 TEF het
## 305 2439   98      0 TEF het
## 306 2440   98      0 TEF het
## 307 2446   97      0 TEF het
## 308 2415   86      0 TEF het
## 309 2418   85      0 TEF het
## 310 2419   85      0 TEF het
## 311 2422   85      0 TEF het
## 312 2423   85      0 TEF het
## 313 2471   81      0 TEF het
## 314 2473   81      0 TEF het
## 315 2474   81      0 TEF het
## 316 2475   81      0 TEF het
## 317 2476   81      0 TEF het
## 318 2477   81      0 TEF het
## 319 2478   81      0 TEF het
## 320 2464   80      0 TEF het
## 321 2465   80      0 TEF het
## 322 2467   80      0 TEF het
```

```
## 323 2430   77      0 TEF het
## 324 1276   66      0 TEF het
## 325 2442   64      0 TEF het
## 326 2454   58      0 TEF het
## 327 2457   58      0 TEF het
## 328 2459   58      0 TEF het
## 329 1326   56      0 TEF het
## 330 2425   19      1 TEF het
## 331 2445   29      1 TEF het
## 332 1970  250      0 TEF het
## 333 1189  232      0 TEF het
## 334  571  227      0 TEF het
## 335  914  211      0 TEF het
## 336 2685  194      0 TEF het
## 337  660  190      0 TEF het
## 338  771  184      0 TEF het
## 339  873  183      0 TEF het
## 340  685  176      0 TEF het
## 341  685  174      0 TEF het
## 342  874  173      0 TEF het
## 343  833  170      0 TEF het
## 344  780  163      0 TEF het
## 345  806  162      0 TEF het
## 346 2296  155      0 TEF het
## 347 1178  154      0 TEF het
## 348  732  152      0 TEF het
## 349  871  151      0 TEF het
## 350 2378  151      0 TEF het
## 351 1201  148      0 TEF het
## 352 2107  148      0 TEF het
## 353 2108  148      0 TEF het
## 354 2114  148      0 TEF het
## 355 1415  147      0 TEF het
## 356  738  146      0 TEF het
## 357  876  141      0 TEF het
## 358  894  137      0 TEF het
## 359  776  136      0 TEF het
## 360 2383  132      0 TEF het
## 361 2190  128      0 TEF het
## 362  810  125      0 TEF het
## 363 1023  120      0 TEF het
## 364 1606  120      0 TEF het
## 365 2096  103      0 TEF het
## 366 2382  101      0 TEF het
## 367 2384  100      0 TEF het
## 368 1105   99      0 TEF het
## 369 2096   98      0 TEF het
## 370 2192   97      0 TEF het
## 371 2539   88      0 TEF het
## 372 2550   88      0 TEF het
## 373 1168   80      0 TEF het
## 374 1172   80      0 TEF het
## 375 1255   72      0 TEF het
## 376 1272   72      0 TEF het
```

```
## 377 2091   70      0 TEF het
## 378 2092   70      0 TEF het
## 379 2094   70      0 TEF het
## 380 2095   70      0 TEF het
## 381 2537   70      0 TEF het
## 382 2540   70      0 TEF het
## 383 1291   66      0 TEF het
## 384 1155   59      0 TEF het
## 385 1327   50      0 TEF het
## 386 2601   50      0 TEF het
## 387 2237   17      1 TEF het
## 388 2218   24      1 TEF het
## 389 2387   18      1 TEF het
## 390 2599   27      1 TEF het
## 391 2728   32      1 TEF het
## 392 1940  158      0 TEF het
```

```
group  <- survival$group
time <- survival$time
status <- survival$status
formula1 <- Surv(time, status == 1) ~ group
test_result <- survdiff(formula = formula1, data = survival, rho = 0)
test_result
```

```
## Call:
## survdiff(formula = formula1, data = survival, rho = 0)
##
##                  N Observed Expected (O-E)^2/E (O-E)^2/V
## group=DBP het  59       13    10.17     0.790     0.928
## group=HLF het  36        4     6.47     0.942     1.038
## group=TEF het 179       13    42.73    20.689    49.854
## group=TKO      96       45    11.67    95.253   116.317
## group=WT       22        0     3.97     3.966     4.220
##
##  Chisq= 126  on 4 degrees of freedom, p= <2e-16
```

```
plot(survfit(formula1), lty=c(1), col=colors1,xlab = "Age (days)", ylab = "Cum Survival Probabil
ity (%)")
legend("right",title="Genotype", legend=c("DBP+/- DKO (n=59)","HLF+/- DKO (n=36)", "TEF+/- DKO
 (n=179)","TKO (n=96)", "WT (n=22)"),col=colors1, lty=1, cex=0.8)
```

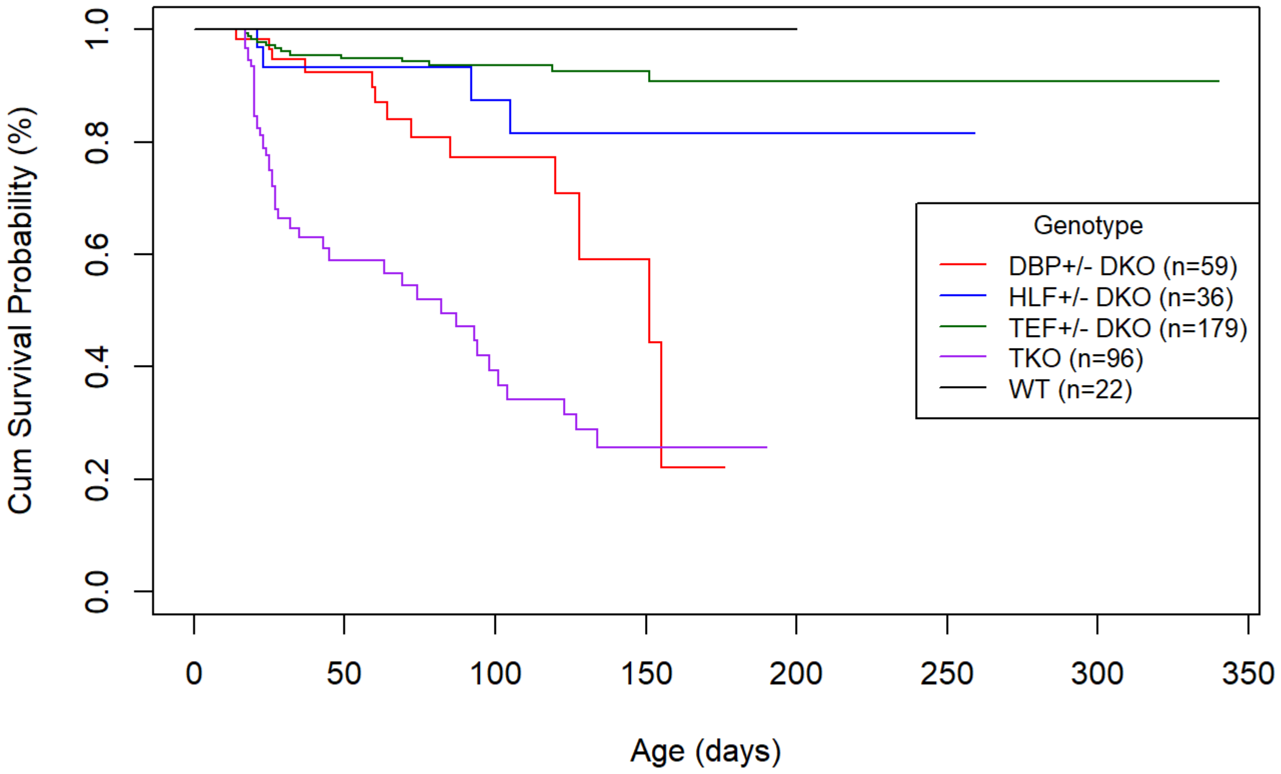
Cum Survival Probability (%)
0.0
0.2
0.4
0.6
0.8
1.0
0
50
100
150
200
250
300
350
Age (days)
Genotype
DBP+/- DKO (n=59)
HLF+/- DKO (n=36)
TEF+/- DKO (n=179)
TKO (n=96)
WT (n=22)
